# Influence of Microenvironment on Mesenchymal Stem Cell Therapeutic Potency: From Planar Culture to Microcarriers

**DOI:** 10.3389/fbioe.2020.00640

**Published:** 2020-06-24

**Authors:** Ang-Chen Tsai, Richard Jeske, Xingchi Chen, Xuegang Yuan, Yan Li

**Affiliations:** Department of Chemical and Biomedical Engineering, FAMU-FSU College of Engineering, Florida State University, Tallahassee, FL, United States

**Keywords:** human mesenchymal stem cells, microcarriers, bioreactors, shear stress, microenvironment, expansion

## Abstract

Human mesenchymal stem cells (hMSCs) are a promising candidate in cell therapy as they exhibit multilineage differentiation, homing to the site of injury, and secretion of trophic factors that facilitate tissue healing and/or modulate immune response. As a result, hMSC-derived products have attracted growing interests in preclinical and clinical studies. The development of hMSC culture platforms for large-scale biomanufacturing is necessary to meet the requirements for late-phase clinical trials and future commercialization. Microcarriers in stirred-tank bioreactors have been widely utilized in large-scale expansion of hMSCs for translational applications because of a high surface-to-volume ratio compared to conventional 2D planar culture. However, recent studies have demonstrated that microcarrier-expanded hMSCs differ from dish- or flask-expanded cells in size, morphology, proliferation, viability, surface markers, gene expression, differentiation potential, and secretome profile which may lead to altered therapeutic potency. Therefore, understanding the bioprocessing parameters that influence hMSC therapeutic efficacy is essential for the optimization of microcarrier-based bioreactor system to maximize hMSC quantity without sacrificing quality. In this review, biomanufacturing parameters encountered in planar culture and microcarrier-based bioreactor culture of hMSCs are compared and discussed with specific focus on cell-adhesion surface (e.g., discontinuous surface, underlying curvature, microcarrier stiffness, porosity, surface roughness, coating, and charge) and the dynamic microenvironment in bioreactor culture (e.g., oxygen and nutrients, shear stress, particle collision, and aggregation). The influence of dynamic culture in bioreactors on hMSC properties is also reviewed in order to establish connection between bioprocessing and stem cell function. This review addresses fundamental principles and concepts for future design of biomanufacturing systems for hMSC-based therapy.

## Introduction

Human mesenchymal stem cells (hMSCs) are a rising candidate for cell therapy and have attracted growing interests due to their ability of immunomodulation and trophic effects beyond tri-lineage differentiation. With thousands of *in vitro* and *in vivo* studies and more than 1000 hMSC-based clinical trials completed or in progress on ClinicalTrials.gov, the potential of hMSCs in therapeutic applications is very promising (Atkinson et al., 2017; Tsuchiya et al., 2019). However, to confirm the effectiveness of hMSCs in cell therapy, late phases of clinical trials require a large amount of cells for transplantation and administration into patients (Yin et al., 2019). In addition, as an immunomodulator, hMSCs exhibit immunoprivileged/immunoevasive properties and can be used in allogeneic therapies, which also demand large-scale biomanufacturing because of the cost of goods (Rowley et al., 2012; Simaria et al., 2014; Zhang et al., 2015; Schnitzler et al., 2016). Due to the limited number of hMSCs acquired from a single donor, *in vitro* expansion under current Good Manufacturing Practices (cGMP) has to be performed to reach pratical cell numbers for dosage requirements in clinical applications (Rojewski et al., 2013; Barckhausen et al., 2016; McGrath et al., 2019). In addition, as an anchorage-dependent cell type, the number of harvested hMSCs should be proportional to the culture surface area in biomanufacturing. Thus, increasing culture surface without sacrificing spacial and labor costs is critical in designing culture vessels in hMSC biomanufacturing. One current technique uses multi-layer vessels designed for cell expansion by stacking layers into one chamber to increase the culture surface. However, these labor-extensive and semi-closed processes require clean room facilities and class-A laminar biosafety cabinets for each step of operation (dos Santos et al., 2013; Martin et al., 2017). Alternatively, automated well-controlled bioreactors provide efficient mixing in a closed system for large-scale expansion in lot size at reduced labor and time, but these automated bioreactors are not readily available (Grayson and Stephenson, 2018; Olsen et al., 2018; Moutsatsou et al., 2019). Among various types of bioreactors that are commercially available, stirred-tank bioreactors with microcarriers are the most commonly used system for scaling-up manufacturing of hMSCs as the microcarriers provide a high surface-to-volume ratio for high density cell culture with a cost of goods reduction ($0.044 per cm^2^) compared to plate stacks ($0.061 per cm^2^) (Simon, 2015). Moreover, microcarrier suspension culture allows real-time cell sampling and off-line analysis for monitoring culture parameters and evaluating critical stem cell properties during expansion. Different feeding strategies, such as batch, fed-batch, and perfusion (dos Santos et al., 2014; Fernandes-Platzgummer et al., 2016), with bead-to-bead transfer can support hMSC stable proliferation under short- and long-term expansion (Panchalingam et al., 2015).

The advantages of microcarrier culture in stirred-tank bioreactors include the scalable design, even cell distribution, homogeneous nutrition and oxygen access, and the timely assessment of medium composition and evaluation of cell properties. Nevertheless, recent studies have shown that microcarrier-expanded hMSCs differ from dish- or flask-expanded cells in size, morphology, proliferation, viability, surface marker, gene expression, differentiation capacity, and secretion of cytokines, which may lead to the alteration of their therapeutic potency (Goh et al., 2013; Hupfeld et al., 2014; Lin et al., 2016; Teixeira et al., 2016). Thus, hMSC properties exhibited in planar culture may not be consistent with microcarrier culture. As seen in our previous studies, these deviations likely result from the altered microenvironment between planar and microcarrier culture in seeding, attaching, expanding, and harvesting, as well as the change of adhesion surface geography and flow-induced dynamic environment (Ma et al., 2016). Therefore, it is important to understand how to optimize bioreactor conditions to maximize hMSC quantity without sacrificing quality and therapeutic potency (Castilla-Casadiego et al., 2020). In this review, influences on hMSC properties from manufacturing parameters in microcarrier bioreactor culture (e.g., discontinuous surface, curvature, microcarrier stiffness, porosity, roughness, coating, and charges) and flow dynamics (e.g., oxygen and nutrient diffusion, shear stress, particle collision, and aggregation) are discussed. Advanced techniques and processes to improve hMSC expansion in microcarrier-based bioreactors are also reviewed.

## The Requirement of hMSCs for Clinical Applications: Quantity and Quality

Originally, hMSCs are isolated from bone marrows, and only occupy 0.001–0.01% of mononuclear cells for healthy adults (Pittenger et al., 1999). hMSCs isolated from other tissues, such as adipose tissues, dermal tissue, dental pump, placenta, and umbilical cords, contain a higher percentage of hMSCs (Fernandes-Platzgummer et al., 2016). For instance, human adipose tissue on average contains 1.2% hMSCs (Fraser et al., 2006), and umbilical cords have 0.3% hMSCs (Wegmeyer et al., 2013). Even though these numbers are 10 to 100 times higher than bone marrow, it is still far below the cell number requirement of one single therapeutic dose (35–350 million hMSCs per dose) in clinical applications (Jossen et al., 2016). Therefore, *in vitro* expansion is necessary to achieve sufficient cells for cell-based therapies. As the demand for hMSC-based therapeutics is increasing exponentially, development of noval biomanufacturing techniques for culture expansion is in urgent need (Olsen et al., 2018).

While most cell therapy products rely on autologous cells for immunologic compatibility (Duijvestein et al., 2010; Honmou et al., 2012), hMSCs provide a possibility in allogeneic therapies as an “off-the-shelf” product (Pittenger and Martin, 2004; Newman et al., 2009; Zhang et al., 2012). According to Robb et al.'s study, 44.05% of clinical trials were targeting allogenic therapies (Robb et al., 2019). Among the 13 commercialized hMSC products, 9 of them use allogeneic approaches (Jossen et al., 2018). In autologous therapies, a rapid expansion of patient-specific cells in a fully closed and automated bioreactor system is important to ensure the product free of cross-contamination with a reasonable yield in minimal production time. The scale-out system can satisfy the need of autologous therapies to parallelly produce personalized products for specific patients (Hourd et al., 2014). In allogeneic therapies, hMSCs manufactured from one or several selective donors are used as a universal drug for multiple patients. The scale-up manufacturing system can reduce the cost of goods to meet the dosage requirement and “off-the-shelf” standard (Pigeau et al., 2018). The importance of the scale-up process is to obtain homogeneous cells from a single batch and eliminate the lot-to-lot variations to manufacture hMSCs as a standardized product. Therefore, the current hurdle for hMSC large-scale expansion in allogeneic therapies is to minimize donor-to-donor variations, reduce bioprocessing fluctuations, and eliminate the cell heterogeneity.

Due to the diversity of characteristics displayed by hMSCs from different sources with various isolation methods, the International Society for Cellular Therapy (ISCT) recommended minimal criteria to define hMSCs: (1) cell adhesion on the plastic surface, (2) specific positive and negative surface markers, and (3) *in vitro* tri-lineage differentiation (Dominici et al., 2006). Although these criteria are helpful to establish the baseline of hMSC characterizations, they cannot be used to evaluate hMSC therapeutic potentials as most of the hMSCs from different research groups satisfy ISCT's criteria while still exhibiting functional variance (Samsonraj et al., 2015; Yuan et al., 2019). For effective cell therapies, hMSC-based products have to be characterized for efficacy, function, and potency. For instance, ISCT reported the test for the levels of vascular endothelial growth factor (VEGF), chemokine (C-X-C motif) ligand 5 (CXCL5), and interleukin (IL)-8 of manufactured MultiStem® (a cellular therapy product of hMSCs) to assess the potency in angiogenesis (Bravery et al., 2013). Similarly, ISCT also required immunological characterization for hMSCs to treat immunological diseases with standardized approaches (Krampera et al., 2013; Galipeau et al., 2016). Although many research groups have reported donor-to-donor variations and tissue source comparisons (Alge et al., 2010; Al-Nbaheen et al., 2013; Menard et al., 2013; Marquez-Curtis et al., 2015; Billing et al., 2016; Isobe et al., 2016), only few studies explored the impact of bioprocessing parameters on hMSC therapeutic potency (Hupfeld et al., 2014; Teixeira et al., 2016; Cunha et al., 2017; Martin et al., 2017).

## Bioprocessing for hMSC Production

Distinct from typical bioprocesses for protein production using organisms, such as *E. coli*, yeast, and Chinese hamster ovary cells, hMSCs themselves are the final products for cell-based therapy. Thus, the focus is on the fold increase in cell number and population doublings rather than the extracellular factor production, although extracellular vesicles have attracted tremendous attentions recently (Phinney et al., 2015; Koniusz et al., 2016). Isolation from non-adherent hematopoietic cells and *in vitro* expansion of hMSCs in planar culture is the initial process, although the resulting cell number can not meet the clinical requirements (Chen et al., 2013; Siddiquee and Sha, 2014; Simaria et al., 2014). Thus, it is necessary to develop new platforms to efficiently expand functional hMSCs on a larger scale to reach sufficient cell number and dosages, which theoretically determines the success of clinical trials (Rowley et al., 2012; Olsen et al., 2018).

**Figure 1:**
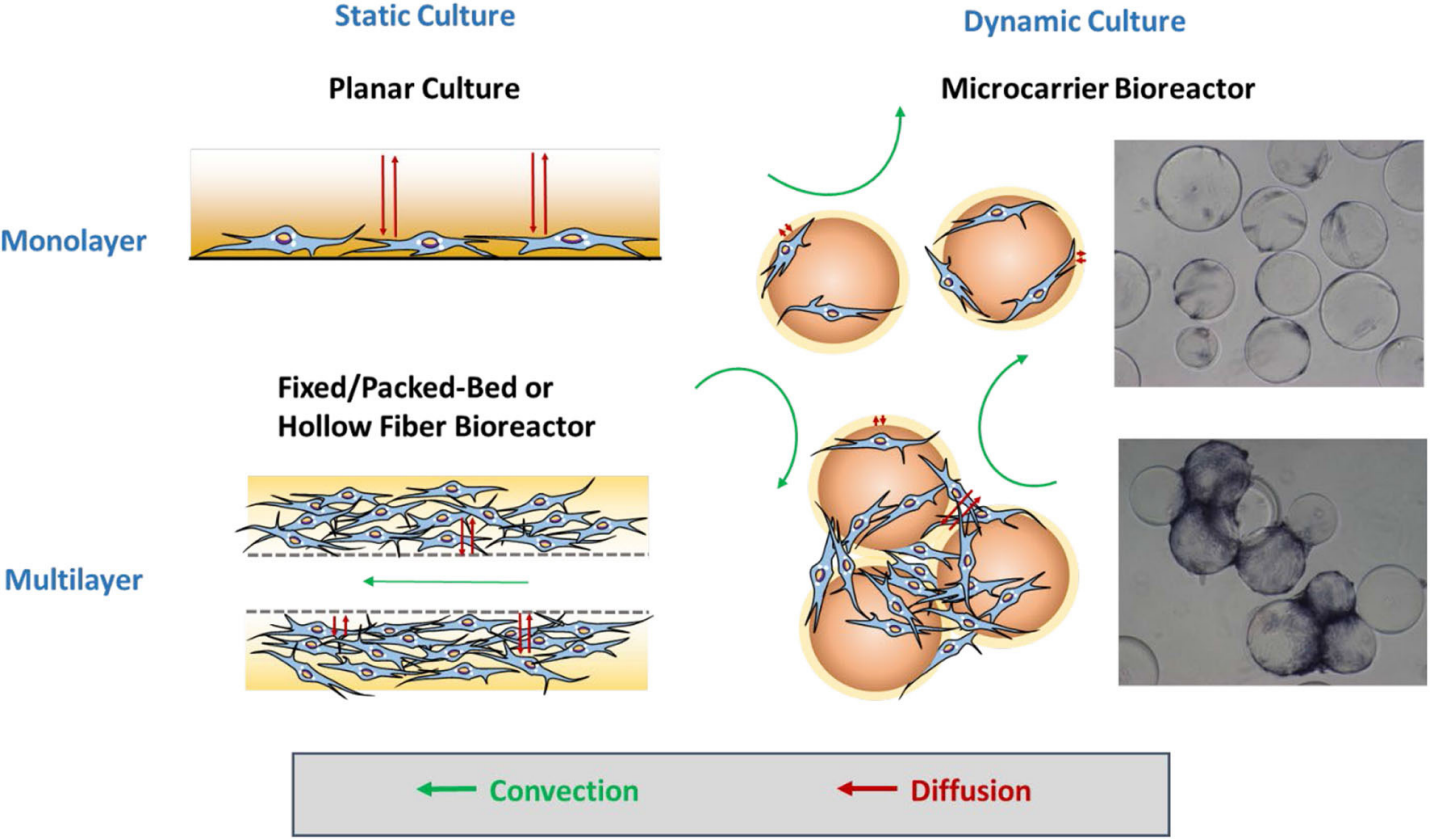
Mass transfer for microcarrier-based culture. For static culture, hMSCs are expanded as monolayers in planar culture or multilayers in the fixed/packed or hollow fiber bioreactor. Mass transfer only relies on diffusion in planar culture, whereas additional convection enhances the mass transfer efficiency in the fixed/packed bed and hollow fiber bioreactor. For dynamic culture in the microcarrier-based bioreactor, hMSCs first attach and grow as monolayers on isolated microcarriers in which convection flows play a more important role in transport. Later, multiple microcarriers are aggregated into a cluster, the internal area of which requires further diffusion for mass transfer like multilayers.

The manufacturing processes under cGMP regulations normally require complete defined serum-free or xeno-free media, effective feeding strategies, automated closed systems, efficient harvest process, cell purification, and cryopreservation methods (Sotiropoulou et al., 2006; Sensebé et al., 2013; Li et al., 2015). The expansion time and population doublings are commonly used as critical quality attributes of the products to evaluate these operational parameters. Knowledge of process control and the parameters acquired from static planar cultures may not be appropriate to directly translate into dynamic bioreactor culture systems without modifications due to the dramatic change in the microenvironment (Sart et al., 2014a; Ma et al., 2016). Therefore, the impacts of bioprocess parameters on hMSC phenotype, cell fate, and therapeutic potency should be investigated systematically. Figure 1 illuminates the types of mass transfer in static culture and dynamic culture in biomanufacturing.

### Multi-Layer Vessels

To manufacture adherent hMSCs, the producion depends on the accessible surface area. Large-scale production of hMSCs requires large surface area, to support efficient cell proliferation while preserving their innate biological properties. Ameliorating from T-flasks, multi-layer vessels, including Nunc™ Cell Factory™ (Thermo Fisher Scientific) and Corning® CellSTACK® (Corning), allow for cell expansion in one chamber by stacking the layers to save the space for incubation, and thus can produce >100 times of cells from a single T-flask. For example, a 40-layer vessel provides a surface of 25,280 cm^2^ which is 144 times of a conventional T-175 flask. The challenge in multi-layer vessel production is the labor-intensive and time-consuming operation. Advanced equipment like automatic robot systems, such as Nunc™ Automatic Cell Factory™ Manipulator System (Thermo Fisher Scientific), are designed for more convenient operations and lowering the risk of contamination.

hMSCs are highly sensitive to biological, chemical, and physical cues in the culture microenvironment, and changes in gene expression and phenotypical markers were observed in planar culture (Bara et al., 2014). Thus, hMSCs cultured in multi-layer vessels exploiting the similar microenvironment to original T-flasks make the bioprocess transfer easier, such as coating surface, seeding density, feeding regimes, and cell harvesting (Ma et al., 2016). So far, multi-layer vessels are commonly used in commercial manufacturing but still with the limited lot size, ranging from 100 to 400 billion cells (Rowley et al., 2012). Furthermore, limitation in characterizations of cell growth and behaviors still exists. For example, real-time observation of cell morphological changes may not be applicable under a regular microscope.

### Fixed/Packed-Bed Bioreactors and Hollow Fiber Bioreactors

Instead of providing a planar surface, fixed-bed/packed-bed bioreactors and hollow fiber bioreactors create a spatial microenvironment for cell growth. In fixed/packed-bed bioreactors, hMSCs are captured within the bed that can be filled with various scaffolds composed of biocompatible polystyrene pellets, glass beads, or fibrous materials to provide large surface area for cell expansion (Zhao et al., 2007; Weber et al., 2010; Osiecki et al., 2015; Tsai et al., 2016). Similarly, in hollow fiber bioreactors (Nold et al., 2013; Mizukami et al., 2018), hMSCs grow in the interstitial space between hollow fibers that were used to mimic blood capillaries (Hanley et al., 2014; Martin-Manso and Hanley, 2015). In these two types of bioreactors, the network of frame structures in hMSC microenvironment not only prevents cells from direct fluidic force, but also supports three-dimensional (3D) cell growth with retention of extracellular matrix (ECM). In addition, 3D architecture better mimics the *in vivo* physiological environment. In the absence of direct exposure to the flow, hMSCs still can benefit from perfused flow, mainly relying on diffusion for mass transfer of nutrients, waste, and oxygen. As a result, cells do not suffer damages from shear stress and physical collisions (Figure 1). Therefore, hMSCs can be cultured in a highly compact 3D system while still reaching a high cell number.

However, fixed/packed-bed bioreactors and hollow fiber bioreactors still have some challenges in the large scale of biomanufacturing: (1) the non-homogeneity of the culture systems; (2) the depletion of nutrition and accumulation of waste when the cell density is too high and the interstitial flow is insufficient to satisfy mass transfer needs; and (3) cell dissociation from a highly condensed 3D clusters (Meuwly et al., 2007; Barckhausen et al., 2016). Other crucial issues include limited potential in scaling-up and inability for continuous long-term culture without enzymatic treatment and passaging. To avoid the depletion of nutrients and accumulation of metabolic waste, perfusion mode can be applied to increase the flow rate according to the real time in culture. A real time analysis of cell state is also possible in fixed bed bioreactor, for example, via oxygen measurements at inlet and outlet and the calculation of cell numbers over oxygen consumption.

### Microcarriers in Spinner Flasks and Stirred-Tank Bioreactors

Microcarriers, designed to replace T-flasks and petri dishes as the adhesion surface for cell growth, have been used for human cell culture since 1967 (Van Wezel, 1967). Most studies on hMSC expansion with microcarriers were conducted in spinner flasks or stirred-tank bioreactors (António et al., 2016), though some other dynamic systems, such as rotating wall vessels or wave motion bioreactors, have also been reported. The apparent advantages for microcarrier suspension culture include the scalable design, homogeneous culture environment, real-time monitoring of cells and medium, and the feasibility to maintain long-term culture via bead-to-bead transfer without enzymatic treatment/passaging (Leber et al., 2017; Rafiq et al., 2018). Moreover, due to the high surface to volume ratio, less culture medium (a main cost driver) is used in hMSC-microcarrier bioprocessing. Therefore,the production cost is reduced. Although this platform has been widely exploited in academia and industry (Badenes et al., 2016a,b), recent studies have shown that cells cultured in spinner flasks or stirred-tank bioreactors are exposed to high and non-homogeneous fluid shear stresses due to the mixing agitation (Ismadi et al., 2014; van Eikenhorst et al., 2014), possibly resulting in reduced hMSC qualities and therapeutic potentials. For example, increasing fluid shear stress during hMSC culture can induce osteogenic and chondrogenic differentiation (Knippenberg et al., 2005; Zhao et al., 2007; Li et al., 2009; Yourek et al., 2010; Schatti et al., 2011). Therefore, application of the slowest agitation rate is always recommended to support both the required mixing of culture and the undifferentiated state of stem cells. Additionally, the fluid flow leads to frequent cell-cell collisions and may result in spontaneous aggregation and clustering, which should be cautiously considered at the late stage of stem cell culture (Caruso et al., 2014; Yuan et al., 2018).

#### Screening of Microcarriers for hMSC Expansion

To date, a wide variety of microcarriers are commercially available. These microcarriers are made of diverse materials, such as polystyrene, dextran, cellulose, gelatin, glass, or decellularized tissue, with different surface properties (Chen et al., 2013; Yu et al., 2017). The size of microcarriers ranges from 100 to 300 μm in diameter, which is large enough for cell adherence. The density of microcarriers is designed between 1.02 and 1.1 g/cm^3^, not only for settling down the microcarriers when changing the medium, but also for reducing input energy of agitation for the submerged suspension. Therefore, screening and comparing these commercial microcarriers to assess hMSC expansion is the initial step.

In addition to the size and density, microcarriers should be evaluated for cell adhesion, expansion, and dissociation. Recently, some commercial microcarriers have been modified from traditional non-porous, uncoated, uncharged surfaces to porous, collagen- or fibronectin-coated, and/or surfaces with positive charge for enhancing cell attachment efficiency (Rafiq et al., 2013a). Based on literature, hMSC attachment on microcarriers can achieve 70% to 90% under the static or dynamic condition in serum-containing medium, whereas the efficiency significantly decreases to 22% to 23% in serum- and xeno-free medium (dos Santos et al., 2011; Timmins et al., 2012). But there is also evidence that hMSCs can grow fast and achieve the same cell density in a xeno-free serum compared to serum-containing culture despite the low adhesion efficiency (Moreira et al., 2020). Currently, most studies on screening microcarriers for bioprocessing only emphasize the seeding efficiency and expansion fold; however, the recovery efficiency or yield is equally critical for the large-scale production (Schnitzler et al., 2012; Timmins et al., 2012; Goh et al., 2013; Rafiq et al., 2016; Moloudi et al., 2019). The cell detachment from microcarriers is not considered as a key factor and has not been optimized in traditional bioprocessing of protein production. Nevertheless, as the final products in cell-based therapies, hMSCs have to be isolated and harvested from these bioreactors. Thus, the detachment efficiency and recovery rate can not be ignored. For example, hMSCs have high expansion fold using Cytodex I microcarriers, but the enzymatic detachment efficiency is low, and it eventually leads to the low cell yield after cell harvest (Weber et al., 2007; Sun et al., 2010; Schnitzler et al., 2012; Timmins et al., 2012; Loubière et al., 2019). Similarly, although macropores provide more surface area for cell expansion and protect cells from direct flows, i.e., shear stress resulted from the eddies at the Kolmogorov scale, the detachment efficiency is generally not applicable for hMSC expansion (Nienow et al., 2016).

In addition to commercial microcarriers, customized microcarriers can be developed to improve the bioprocessing for hMSC attachment, proliferation, and harvest as well as maintaining their phenotype and therapeutic potency. For example, hydrogel-based microcarriers can improve cell attachment and harvesting efficiency (Chui et al., 2019). Thermo-responsive surfaces on microcarriers have been developed to improve harvest efficiency without enzymatic treatment (Yang et al., 2010; Song et al., 2016; Yuan et al., 2018). Biodegradable, implantable, or enzyme-dissolvable microcarriers can avoid the dissociation or separation step during cell harvesting (Sart et al., 2009; Sun et al., 2010; Park et al., 2013; Shekaran et al., 2016; Confalonieri et al., 2017; Wang et al., 2017; Rodrigues et al., 2019). Microcarriers coated with chemically-defined polymers can better control culture systems and increase the reproducibility (Krutty et al., 2019). Hollow microcarriers provide inner surface for cells to grow and are able to protect hMSCs from direct flow damage (YekrangSafakar et al., 2018). Magnetic microcarriers can speedily settle down when changing medium, and are easily separated from cells after enzymatic treatment to improve cell yield (Lin et al., 2014). Modification of surface charge may be another approach to enhance hMSC attachment or detachment (Rafiq et al., 2016). However, each method should be carefully reviewed and modified for large-scale bioprocessing as most studies were conducted at the laboratory scale. For example, when using the microcarriers with a thermo-sensitive surface, the temperature should be well-controlled throughout bioprocessing. To simplify the harvesting process, novel dispersible and dissolvable porous microcarriers have also been reported recently (Yan et al., 2020).

#### Agitation

Agitation in stirred-tank bioreactors provides the driving force to generate convective flow, and proper agitation is needed for homogenization of culture microenvironment, dispersion of gas and nutrients, optimal mixing, reduction of the laminar boundary layer, and to increase the convective mass transfer coefficients, all of which are important in MSC bioprocessing. For bioreactors at different scales and with different microcarriers, the operation of agitation should be evaluated for hMSC adhesion, expansion, and dissociation.

To maximize seeding efficiency with an even cell distribution, it is recommended to utilize intermittent agitation and reduced initial working volume. For example, the use of intermittent agitation (3 min agitation at 60 rpm followed by no agitation for 27 min) showed 1.5 to 2-fold higher attachment efficiency than the continuous agitation (60 rpm) in the first 24 h for hMSC expansion on CultiSpher-S microcarriers (Yuan et al., 2014).

**Figure 2:**
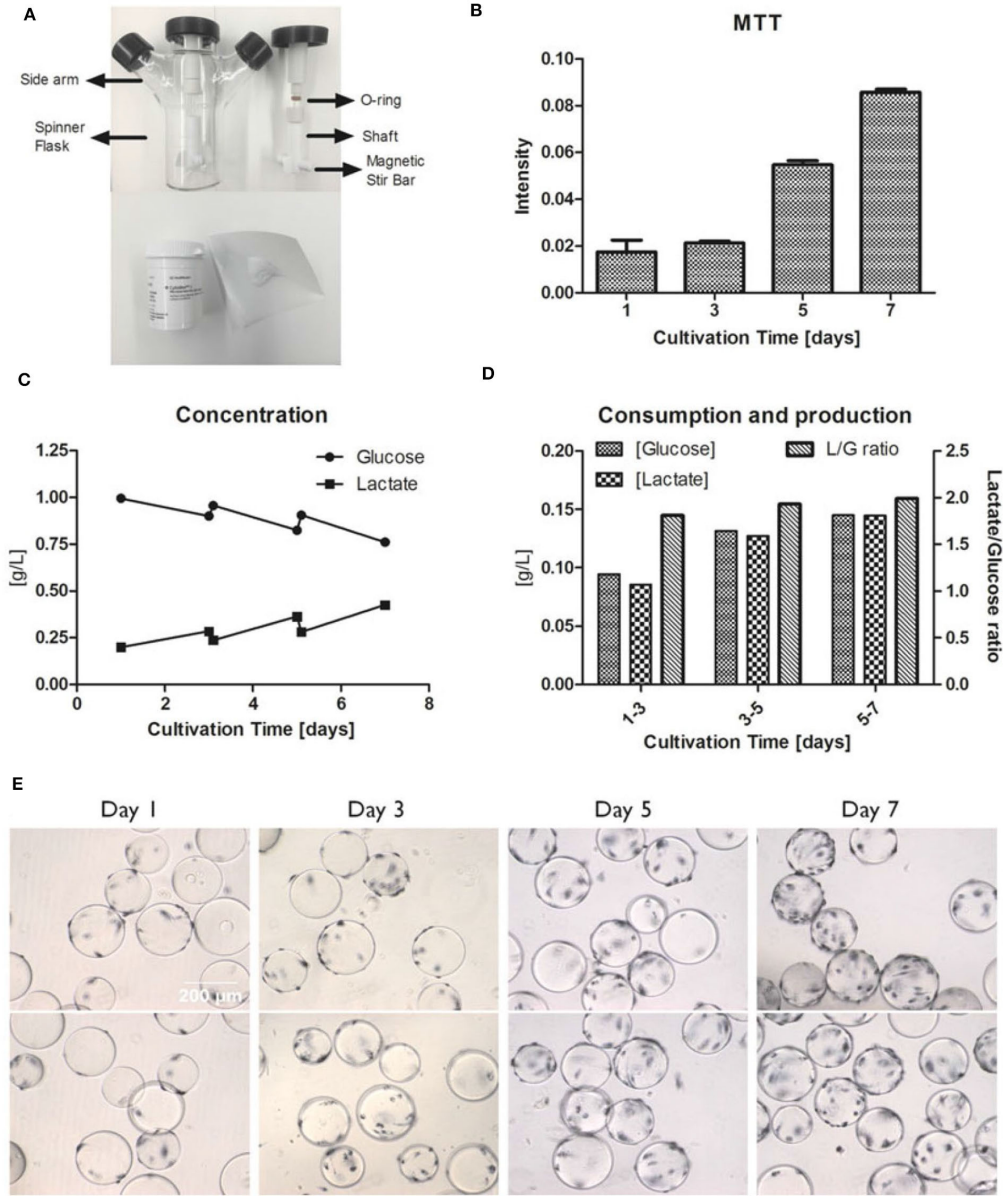
Example of hMSC growth in microcarrier-based spinner flasks. **(A)** Spinner flask bioreactor and internal components (top). Cytodex I microcarriers (bottom). **(B)** hMSC proliferation measured by MTT intensities at day 1, 3, 5, and 7. **(C)** Concentration of glucose and lactate in the media over a 7-days culture period. **(D)** The glucose consumption and lactate production with the lactate/glucose ratio over a 7-days culture period. **(E)** MTT staining of hMSCs on Cytodex I at day 1, 3, 5, and 7. The spinner flask was operated at 30 rpm. Reference: Tsai and Ma (2016). Copyright was permitted.

To determine the agitation speed for hMSC expansion, N_S1_ and N_S1u_ were introduced to represent the criteria of agitation for microcarrier suspension culture. N_S1_ stands for the minimum impeller speed that fully suspends the microcarriers, and N_S1u_ represents the agitation speed that merely allows microcarriers moving along the bottom of bioreactors (Schirmaier et al., 2014; Jossen et al., 2016). It is noted that N_S1_ and N_S1u_ are highly dependent on working volume, microcarrier type and concentration. For instance, Kaiser et al. found no significant difference in expansion when culturing adipose tissue-derived hMSCs at 49, 60, and 82 rpm on Hillex® II and ProNectin®-F-coated microcarriers, and it is possible that the agitation speed is still within the optimal N_S1_ range (Kaiser et al., 2013). A follow-up study tested a broader range of agitation speeds (25–120 rpm) in spinner flasks with 100 mL working volume, and the results showed that hMSCs reached the highest expansion (117-fold) at 49 rpm, and only 71-fold and 19-fold increase were observed at 25 and 120 rpm, respectively (Jossen et al., 2016). Another study showed that hMSCs cultured on CultiSpher-S microcarriers at 60 and 90 rpm had slightly higher expansion (5.5-fold) compared to the culture at 115 rpm (4.3-fold) after an 8-days culture (Yuan et al., 2014). Undoubtedly, optimized agitation speed is critical for hMSC expansion, because higher agitation speed may inhibit hMSC growth due to cell exposure to high shear stress, while low agitation speed cannot fully suspend microcarriers, leading to microcarrier clustering (Jossen et al., 2014; Takahashi et al., 2017). Furthermore, the agitation speed has a linear correlation to the average fluid shear stress in spinner flasks (Ismadi et al., 2014). Based on this observation, Nienow reported a method to detach hMSCs from Plastic P102-L microcarriers with a short time of exposure to the high agitation (220 rpm), which generates high shear stress and reduces Kolmogorov scale (Nienow et al., 2014). Our study also showed the hMSC expansion when cultured on Cytodex-1 microcarriers (Tsai and Ma, 2016). The 30 rpm agitation was observed to support active metabolic activity and glucose consumption, which is usually associated with high cell growth rate (Figure 2).

It needs to be noted that the agitation speed using rpm cannot be compared among different culture systems. It is better to use Reynolds number or tip speed [m/s], with the knowledge of the exact reactor set up and geometry. In particular, to transfer the knowledge from spinner flasks to large stirred tank bioreactors, a dimensionless number, such as Reynolds number should be used.

Considering the high and non-homogeneous shear stress in the spinner flasks or stirred-tanks, several bioreactor geometries have been designed to reduce the effects of shear stress. For example, an indentation on the bottom of the spinner flasks can prevent microcarriers from accumulation and clustering below the impeller where the lowest flow velocity occurs (Kaiser et al., 2013). Side baffles can convert the rotational flow to radial flow and axial flow to enhance the microcarrier suspension (Nienow, 2006; Rafiq et al., 2013a). The dimensions, shapes, locations, and orientations of impellers exhibit specific different hydrodynamics, in terms of microcarrier suspension and shear stress exposure (Nienow, 2006; Ismadi et al., 2014; Collignon et al., 2016). A pitched-blade impeller design has been commonly used in stirred-tank bioreactors for hMSC expansion (Goh et al., 2013; Rafiq et al., 2013a; dos Santos et al., 2014; Siddiquee and Sha, 2014; Fernandes-Platzgummer et al., 2016).

#### Spinner Flasks vs. Stirred-Tank Bioreactors

Compared to stirred-tank bioreactors, spinner flasks (Figure 2) also create dynamic environment due to stirring and can be easily set up in a regular incubator for process optimization and characterization. The available volume for commercial spinner flasks are from 25 mL to 36 L, with 50–250 mL commonly selected for lab use (Eibes et al., 2010; dos Santos et al., 2011; Hewitt et al., 2011; Kaiser et al., 2013; Caruso et al., 2014; Hervy et al., 2014; Schirmaier et al., 2014; Heathman et al., 2015; Petry et al., 2016; Tsai and Ma, 2016). Although spinner flasks have comparable design to stirred-tank bioreactors, there still exist some differences (Table 1). Volumetric productivity and fold increase can be used to evaluate hMSC expansion. Rafiq et al. (2013a) demonstrated that 5 L stirred-tank bioreactors can support slightly higher expansion fold (6.02 and 7.02) of hMSCs than 100 mL spinner flasks (3.66 and 5). Similar trends were also reported by Goh et al. using 1 L stirred-tank bioreactors in comparison with 100 mL spinner flasks (Goh et al., 2013). Though, scaling-up normally deals with non-homogeneous quality and a lower yield because of extensive handling and mass transfer limitations (Hernandez, 2016), there are considerations in bioprocessing for choosing stirred-tank bioreactors rather than spinner flasks. As an open system, spinner flask is suitable for laboratory expansion and cannot be scale-up with processing control and automation. Moreover, hMSC products of clinical grade have requirements for product standardization and robustness, which can only be achieved through closed bioreactor systems. Due to a better control on culture environment (e.g., pH, oxygen), improved hMSC expansion can be observed in stirred-tank bioreactors compared to spinner flasks.

**Table 1:** Comparison of spinner flask and stirred-tank bioreactor.

**Parameters**	**Spinner flask**	**Stirred-tank bioreactor**
Impeller type	Stir bar and impeller	Various types (Mirro and Voll, 2009)
Agitation mixing direction	Radial	Radial and/or axial
DO measurement	None for most cases; Some spinner flasks, e.g., from Presens can monitor DO level (Demuth et al., 2016)	DO probe
DO control	None	O_2_ input and gas-sparging. Use a proportional–integral–derivative (PID) controller which switches on the aeration with air or pure oxygen, several aeration strategies can be performed, e.g., gas bubbles, membrane aeration etc. (Levinson et al., 2015)
pH monitor	None for most cases; Some spinner flasks, e.g., from PreSens can monitor pH level (Demuth et al., 2016)	pH probe
pH control	Incubator CO_2_ concentration	Addition of acid and base; Or CO_2_ gassing (Hoshan et al., 2019)
Scalability	Small	Medium to large
Closed system	No	Can be (Levinson et al., 2015)
Inner pressure	As ambient	Positive pressure (Wilson and Kowol, 1994)
Condenser	None	Equipped

In spinner flasks, pH value is regulated by the ambient CO_2_ concentration in the incubator and there is no control on dissolved oxygen (DO). Conversely, most large-scale stirred-tank bioreactors can balance the pH value by adding diluted acids or bases, and compensate the DO level by adjusting partial oxygen tension input or turning on the gas sparging. Another discrepancy is that large-scale stirred-tank bioreactors are usually equipped with a condenser to avoid medium evaporation, which is alleviated by the humidified ambiance in the incubator (95% relative humidity at 37°C) for spinner flasks. Positive pressure in the head space also prevents evaporation in bioreactors. Other differences, including the extra perfusion system to attenuate media variation, the baffles on the side wall to enhance vertical mixing, and the homogenization at different scales of working volume with different impeller geometry, may further promote hMSC production in stirred-tank bioreactors.

The challenges in large scale manufacturing of hMSCs lie in the mixing of circulation system and non-homogeneous cell quality. The process parameters from several studies for scale-up production of hMSCs are summarized in Table 2. Only a few cases achieved successful expansion of hMSCs in bioreactors at large scale. For example, Schirmaier et al. reported that production of 1 × 10^10^ hMSCs can be achieved at a harvest density of 3 × 10^5^ cells/mL in a 50-L single-use stirred-tank bioreactor (CultiBag® STR 50 L, Sartorius Stedim Biotech) with 35 L working volume (Schirmaier et al., 2014). Similarly, Lawson et al. reported that 1.28 × 10^10^ hMSCs were harvested at the density of 2.56 × 10^5^ cells/mL in a 50-L single-use stirred-tank bioreactor (Mobius® 50 L, MilliporeSigma) with 50 L working volume (Lawson et al., 2017). In spite of the lower harvest cell density in large scale bioreactors (Table 2), comparable volumetric productivity in UniVessel® SU 2 L bioreactors (Sartorius Stedim Biotech) and CultiBag® STR 50 L bioreactors was also reported (Schirmaier et al., 2014). The commonly used scale up criteria include similar power input per volume, gas flow rate per reactor volume (vvm), and oxygen transfer coefficients (Xu et al., 2017). The correspondingly adjusted operational parameters, such as the geometry of vessels and impellers, agitation speed, microcarrier concentration, working volume, aeration (type and vvm), feeding strategies, and seeding/harvesting procedures, all impact culture microenvironment and thus cell production (Castilla-Casadiego et al., 2020).

**Table 2:** Case studies of microcarrier-based bioreactor systems for human mesenchymal stem cell expansion.

**Cell source**	**Working volume (mL)**	**Vessel model**	**Micro-carrier type**	**Medium**	**Agitation (rpm)**	**Tip speed (cm/s)**	**Fold increase**	**Time (day)**	**Seeding density (10^**5**^/mL)**	**Harvest density (10^**5**^/mL)**	**Glucose consu. (pmol/cell/day)**	**Lactate product. (pmol/cell/day)**	**Quality test (gene or protein)**	**Differentiation**	**References**
hAD-MSC	100	Spinner flask; Single use	Hillex II; ProNectin-F (P)	EGM-2 MV	49 60 82	10.9 13.0 17.8	16.3–18.3 (Hillex) 26.4–31.4 (P)	6	0.15	–	–	–	–	–	(Kaiser et al., 2013)
hAD-MSC; hBM-MSC	80	Spinner flask	Cultispher-S; Non-porous plastic	MesenPRO RS; StemPro MSC Xeno-Free	40	–	14 ± 7 (AD) 18 ± 1 (BM)	14	0.1	1.4 ± 0.5 (AD) 2 ± 0.2 (BM)	12 ± 2 (AD) 10 ± 1 (BM)	19 ± 2 (AD) 15 ± 1 (BM)	CD31 CD73 CD80 CD90 CD105 HLA-DR	Tri-lineage differentiation	(dos Santos et al., 2011)
hPL-MSC	500	Culti-Bag; Single use	Cytodex-1; Cytodex-3; Cultispher-S; FACT; ProNectin Collagen	DMEM + 20% FBS	–	–	14.9 ± 1.2 (Cultispher-S) 15.7 and 16.3 (5% O_2_)	7	–	–	–	–	CD44 CD45 CD73 CD90 CD105 CD146	Tri-lineage differentiation	(Timmins et al., 2012)
hPL-MSC	100	Spinner flask	Cytodex-3	DMEM + 10% FBS	50 (type 1) 30 (type 2)	11.7 (type 1) 8.6 (type 2)	2.0–11 (type 1) 3.0–20 (type 2)	8.0–10	0.15–0.75	0.6–3.8 (type 1) 0.7–3.8 (type 2)	–	–	–	–	(Hewitt et al., 2011)
hBM-MSC	50	Cell-Spin	Culti-spher S	MesenPRO; DMEM + 10% FBS	30	–	8.4 ± 0.8	8	0.5	4.2	5.4 ± 0.3	10.3 ± 0.9	CD73 CD90 CD105	Osteo-genesis; adipo-genesis	(Eibes et al., 2010)
hUC-MSC	100	Spinner flask	Plastic Plus (screen micro-carriers)	DMDM + 10% hPL	40	–	16.4 (Male) 13.8 (Female)	7 (Male) 6 (Female)	0.16 (Male) 0.38 (Female)	2.6 (Male) 5.3 (Female)	–	–	CD11b, CD19, CD34, CD45, CD73, CD90, CD105; HLA-DR; CFU-F	Tri-lineage differentiation	(Petry et al., 2016)
hAD-MSC	100 1,500	Spinner flask; Biostat Culti-Bag	Polystyrene with 2 different densities and sizes	Lonza medium + 5% FBS	25, 43, 49, 63, 90, 120	–	71.4, 79.6 117, 97.4 28.5, 19.4 6.59 (Cultibag)	7 (49 rpm) 9	0.108 (SF)	12.5 ± 0.05 (49 rpm)	1.08–5.07	2.43–8.82	CD14, CD20, CD34, CD45, CD73, CD90, CD105	–	(Jossen et al., 2016)
hBM-MSC	50	Spinner flask	Cytodex 3	αMDM + 15% FBS	50	–	3.9	7	1.25	4.82 ± 1.18	1.86	4.04	CD13, CD14, CD29, CD31, CD45, CD49e, CD90, CD105, CD146; HLA-DR	Osteo-genesis; adipo-genesis	(Caruso et al., 2014)
hF-MSC	100	Spinner flask; Biostat B-DCU	Cytodex 3	DMEM or αMEM + 10% FBS	30 (SF) 60–80 (Bio-stat)	–	10 (SF DMEM) 10 (SF αMEM)	8, SF DMEM 6–7, SF αMEM	0.5	5 (SF DMEM) 5.1 (SF αMEM) 10.8 (SF αMEM)	12 ± 1.2 (SF DMEM) 4.3 ± 1.4 (SF αMEM)	23.7 ± 5.3 (SF DMEM) 7.5 ± 0.2 (SF αMEM)	CD34 CD73 CD90 CD105	Tri-lineage differentiation	(Chen et al., 2015)
hBM-MSC	100	Spinner flask	Non-porous Plastic P-102L	DMEM + 10% FBS; PRIME-XV™ SFM		Paddle 50 mm in diameter							CD34 CD73 CD90 CD105 HLA-DR	Tri-lineage differentiation	(Heathman et al., 2015)
hBM-MSC	2,200 250	Verti-cal Wheel (PBS); Biostat Qplus stirred-tank	Corning Synthemax II	MesenCult™-XF	17 (PBS) 40–45 (Bio-stat)	–	12 (PBS) 11 (Biostat)	14	0.25	3 (PBS) 2.8 (Biostat)	6.72 ± 1.92	13.92 ± 1.68	CD34, CD44, CD73, CD90, CD105, CD166 HLA-DR	Tri-lineage differentiation	(Sousa et al., 2015)
hBM-MSC	35–45	Spinner flask; Single use	Corning Syn-themax II	Mesen-cult XF (M); Stempro hMSC (S)	30 rpm every 2 h for 15 min	–	5 (M) 7 (S) 3 × 10^7^ (M) 10,000 (S)	5 42 (M) 26 (S)	0.21–0.29	–	–	–	CD14 CD45 CD73 CD105	Osteo-genesis; adipo-genesis; chondro-genesis	(Hervy et al., 2014)
hF-MSC	100 1,000	Spinner flask; Biostat B-DCU	Cytodex-1 Cytodex-3 Culti-spher GL HyQ-sphere P102-L	DMEM + 10% FBS	30 (SF) 50 (Bio-stat)	–	13.6 (SF) 12 (Biostat)	11 (SF) 8 (Biostat)	0.5	6.8 ± 0.1 (SF) 6 ± 0.2 (Biostat)	5.5 (Biostat) 12.5 (Planar)	10 (Biostat) 30.5 (planar)	CD34, CD73, CD90, CD105 STRO-1 CFU-F; ALP; Calcium deposition	Osteo-genesis	(Goh et al., 2013)
hBM-MSC	2,500	Biostat B Plus (Bio); Spinner flask	Nonporous Plastic P-102L	DMEM 10% FBS	75 (Bio) 30 (SF)	62.8 (Bio) 9.4 (SF)	7.02 and 6.02 (Bio) 3.66 and 5 (SF)	12 (Bio) 12 (SF)	0.24	1.68 and 1.44 (Bio) 1.1 and 1.5 (SF)	8.0–14 (Bio) 8.0–11 (SF)	22–28 (Bio) 23–28 (SF)	CD14, CD19, CD34, CD45, CD73, CD90, CD105 HLA-DR	Tri-lineage differentiation	(Rafiq et al., 2013b)
hBM-MSC	3,000	Mobius Cell-Ready Single use	Cytodex 1 Cytodex 3 collagen-Hillix; Culti-spher G, S	DMEM 10% FBS	25–35	–	40	12	0.05 by calculation	>2	–	–	CD11b, CD14, CD19, CD34, CD44, CD45, CD73, CD79α, CD90, CD105, CD106, CD146, CD274	Osteo-genesis; adipo-genesis	(Kehoe et al., 2013)
hBM-MSC	200 2,000	Spinner flask; Mobius Cell-Ready; Single use	Collagen-coated Solohill C102-1521	DMEM 10% FBS	30 (SF) 25 (1 L) 40 (2 L)	–	5.2	5	0.2 (125 mL) 0.3 (3 L)	0.75	2.7 × 10^−9^ g/cell/day	1.9 × 10^−9^ g/cell/day	CD105 CD14 CD19 CD44 CD90 (protein and gene)	Adipo-genesis	(Schnitzler et al., 2012)
hAD-MSC	100 2,000 35,000	Spinner flask; UniVessel SU; Culti-Bag	Pro-Nectin F-COATED	Specialmedium (Lonza, USA) 5% FBS	60 (100 mL) 100–140 (2 L) 50–66 (35 L)	13 (100 mL) 28.3–39.6 (2 L) 37.4 (35 L)	58.4 ± 12.4 (100 mL) 35.4 ± 0.4 (2 L)	7	0.05–0.1	6.1 ± 1.9 (100 mL) 2.7 ± 0.2 (2 L) 3.1 (35 L)	–	–	CD34 CD45 CD73 CD90 CD105	–	(Schirmaier et al., 2014)
hAD-MSC hBM-MSC	800	Spinner flask; to Bioflo 110	Nonporous plastic (SoloHill)	StemProMSC SFM Xeno-Free	40	–	3 (AD) 7 (BM)	(4) + 7	0.5	0.57 ± 0.2 (AD) 1.3 ± 0.1 (BM)	12.0–13	23–25	CD31, CD73, CD80, CD90, CD105 HLA-DR	Tri-lineage differentiation	(dos Santos et al., 2014)
hAD-MSC	3,750	Bio-BLU 5c; Single use	Polystyrene P-221-040 (PS); collagen C102-1521	ATCC basal medium	25 (PS) 35 (Collagen)	–	7 (PS) 14 (Collagen)	18 (PS) 16 (Collagen)	0.05 (PS) 0.175 (Collagen)	0.39 (PS) 2.4 (Collagen)	–	–	CD44 CD90 CD105 Oct3/4 Sox2	Osteo-genesis; adipo-genesis	(Siddiquee and Sha, 2014)
hBM-MSC	2,400 50,000	Mobiu; Single use	Pall collagen-coated MCs	αMDM 10% PL	For 50 L, 64 for 4 h 75–85 95–100	–	64 (3 L) 36 (50 L)	9 (3 L) 11 (50 L)	0.0625 (3 L) 0.05 (50 L)	4 (3 L) 1.9 (50 L)	–	–	CD11b, CD14, CD19, CD34, CD44, CD45, CD73, CD79a, CD90, CD105 HLA-DR	Tri-lineage differentiation	(Lawson et al., 2017)
hPDCs	80	Spinner flask	Cultispher-S	DMEM 10% FBS	30	–	3.2 ± 0.64	12	0.25	0.8	500–1,000	1,000–2,000	CD14, CD20, CD34, CD45, CD73, CD90, CD105	Tri-lineage differentiation	(Gupta et al., 2019)
hPDCs	80	Spinner flask	Cultispher-S	DMEM 10% hPL or 10% FBS	–	–	5.2 ± 0.61 (hPL) 2.7 ± 0.22 (FBS)	10	0.25	1.3 (hPL) 0.675 (FBS)	500–1,300	1,000–2,000	CD14, CD20, CD34, CD45, CD73, CD90, CD105	Tri-lineage differentiation	(Gupta et al., 2018)

*MSC, mesenchymal stem cells; hAD-MSC, human adipose tissue-derived MSC; hBM-MSC, human bone marrow-derived MSC; hPL-MSC, human placenta-derived MSC; hUC-MSC, human umbilical cord-derived MSC; hF-MSC, human fetal bone marrow-derived MSC; hPDCs, human periosteum-derived MSC; FBS, fetal bovine serum; hPI, human platelet lysate; SF, spinner flask; CFU-F, colony-forming units-fibroblast; ALP, alkaline phosphatase*.

## Characterization of hMSCs in Microcarrier Suspension Culture

hMSCs cultured on microcarriers are exposed to a significantly different microenvironment from planar culture, therefore it is still unclear how much knowledge obtained from planar culture can be translated to microcarrier culture. Most studies mainly reported the increased folds in cell number, the expression of surface markers, colony formation ability, and differentiation capability of microcarrier-expanded hMSCs. However, these criteria only represent the minimal properties of hMSCs and do not indicate their therapeutic potency. In addition, bioprocessing parameters, such as type of microcarriers, controlled agitation, and culture scale, all may influence hMSC properties. Thus, understanding how the microcarrier culture acts on cellular behaviors and how these process parameters change therapeutic potency are beneficial for hMSC-based therapies and the associated biomanufacturing (Table 3).

**Table 3:** Alteration of hMSC properties from planar culture to microcarrier-based bioreactor culture.

**hMSC Characteristics**	**Microcarrier-base bioreactor vs. planer**	**Therapeutic perspective**	**References**
Expansion	Extended lag phase; Comparable/lower proliferation rate; Longer doubling times; Stable chromosome	Support long-term culture and large-scale expansion; satisfy clinical cell dosage requirements	Schatti et al., 2011; Hanley et al., 2014; Hupfeld et al., 2014; Martin-Manso and Hanley, 2015; Yu et al., 2017
Phenotype	Stable ISCT criteria; Negative for CD349 in UC, AM, Placenta; CD146 ↓ in UC, AM and BM; HLA-DR↑ in BM	Meet ISCT's minimal criteria, while certain markers have variations	Timmins et al., 2012; Hupfeld et al., 2014; Collignon et al., 2016; Petry et al., 2016; Lawson et al., 2017
Differentiation potential	Osteogenic differentiation ↑; Adipogenic differentiation ↓; Chondrogenic differentiation ↑	Lineage commitment via modification of microcarrier surface properties.	Aggarwal and Pittenger, 2005; Sun et al., 2010; Hervy et al., 2014; Hupfeld et al., 2014; Kang et al., 2015; Panchalingam et al., 2015
Migration ability	Cell size ↓; CXCR4 ↑	Improve MSC homing after transplantation	Levato et al., 2015; Yu et al., 2017
Secretory function (Immunomodulation, Angiogenesis and neuroprotection)	IL-6 ↑; IL-8 ↑; CXCL5 ↑; Cystatin C ↑; GDN ↑; Galectin-1 ↑; PEDF ↑; BDNF ↑; IGF-1 ↑; VEGF ↑; IL-1ra ↑; SDF-1a ↑; bFGF ↑; M-CSF ↑; NGF ↑; MCP-1 ↑; HGF ↑	Maintain or improve anti-inflammation and immunomodulation for T cells and macrophages after transplantation, enhance therapeutic effects in neurological disease.	Fernandes-Platzgummer et al., 2016; Lin et al., 2016; Petry et al., 2016; Teixeira et al., 2017

*UC, umbilical cords; AM, amniotic membrane; BM, bone marrow; CXCR4, C-X-C chemokine receptor type 4; BDNF, brain-derived neurotrophic factor; CXCL5, C-X-C motif chemokine 5; GDN, glia-derived nexin; HGF, hepatocyte growth factor; IGF-1, insulin-like growth factor 1; IL-1ra, interleukin 1 receptor antagonist; IL-6, interleukin 6; IL-8, interleukin 8; MCP-1, monocyte chemotactic protein-1; M-CSF, macrophage colony-stimulating factor; NGF, nerve growth factor; PEDF, pigment epithelium-derived factor; SDF-1a, stromal-derived-factor-1; VEGF, vascular endothelial growth factor*.

### Expansion and Proliferation

In microcarrier culture of hMSCs, a long lag phase in cell proliferation is commonly observed (Eibes et al., 2010; Sun et al., 2010; dos Santos et al., 2011; Hewitt et al., 2011; Goh et al., 2013), suggesting that the cells need longer time to adjust themselves to the culture environment in bioreactors compared to planar culture. The microcarrier culture systems have not been maturely developed for hMSC expansion yet, and may not have reached the maximum expansion potential. hMSCs grow faster in planar culture than microcarrier culture, showing higher proliferation rates and lower doubling times (Sun et al., 2010; Goh et al., 2013). However, when only considering the exponential phase, microcarrier culture can have comparable or increased growth rate after optimization (Sun et al., 2010). Moreover, long-term culture can be achieved by bead-to-bead transfer in microcarrier bioreactors. Hence, as long as the lag phase can be reduced, microcarrier bioreactors are suitable for the production of hMSCs within a comparable time to planar culture.

In planar culture, cell density is typically expressed as cells/cm^2^. In microcarrier culture, cell density can also be described by cells/mL. Using vendor's information on the surface area per unit mass of microcarriers (cm^2^/g), growth area at a certain microcarrier concentration in the vessel can be calculated. Loss of microcarriers may occur in each step of washing, transferring, medium changing, and harvesting. Extra microcarriers, about 5–10%, can be added for compensation if needed. With online systems, e.g., impedance spectroscopy, cell biomass on the microcarriers can be determined at real time with no loss due to sampling.

### Phenotype Characterization and Colony-Forming Unit-Fibroblast (CFU-F)

Current studies on microcarrier expansion of hMSCs usually provide results for the expression of positive surface markers (CD73, CD90, and CD105) and negative surface markers (CD45, CD34, CD14 or CD11b, CD79α or CD19, and HLA-DR) along with *in vitro* tri-lineage differentiation capacity (dos Santos et al., 2011; Goh et al., 2013; Kehoe et al., 2013; Rafiq et al., 2013a; Caruso et al., 2014; Siddiquee and Sha, 2014; Jossen et al., 2016; Petry et al., 2016). It is well-known that surface marker expression can be different for hMSCs due to various donors and tissue sources. Hupfeld et al. (2014) manifested that the bioprocessing (planar vs. microcarrier) may affect the expression of hMSC phenotypic marker CD349 (frizzled 9) in three different donors. In particular, flask-expanded hMSCs derived from amniotic membrane and umbilical cord positively expressed CD349, whereas microcarrier-expanded hMSCs did not. And CD349^−^ hMSCs derived from placenta can effectively recover blood flow after vascular occlusion in a mouse model rather than CD349^+^ hMSCs (Tran et al., 2011), suggesting that microcarrier-expanded hMSCs may have higher capacity of arteriogenesis and angiogenesis (Hupfeld et al., 2014). Other surface markers, including CD136, CD143, CD146, and CD200, were expressed inconsistently in Hupfeld's study. Interestingly, Shekaran et al. reported that a significant decrease of CD146 expression, known as a pericyte- and endothelial-specific marker (Shi and Gronthos, 2003) or a marker of hMSC multi-potency (Russell et al., 2010), in microcarrier-expanded fetal hMSCs compared to planar culture (Shekaran et al., 2015). Moreover, the expression of CD105 was reported to decrease from more than 90% to 85.9 ± 7.9 and 86.7 ± 2.4 for Cultispher-S and plastic microcarriers, respectively (dos Santos et al., 2011; Mizukami et al., 2018).

In addition, microcarrier-expanded hMSCs were reported to have higher expression of early osteogenic gene markers, such as RUNX2, ALPL, and Osterix/SP7, and late osteogenic marker IBSP during osteogenic differentiation, indicating that hMSCs from microcarrier culture may favor osteogenic lineage commitment (Shekaran et al., 2016). Similarly, microcarrier culture was reported to up-regulate RUNX2, ALP, COL1, and SOX9 gene expression of placenta-derived hMSCs compared to planar culture (Tseng et al., 2012). Microcarrier-expanded hMSCs were also reported to have higher gene expression of crucial chondrogenic transcriptional regulators, such as SOX9, SOX5, and SOX6, as well as the chondrogenic ECM marker COL2A1, suggesting that microcarrier culture may augment chondrogenic commitment (Lin et al., 2016). In addition, hMSCs harvested from microcarriers were found to have slightly lower CFU-F number in comparison with hMSCs from monolayers (Goh et al., 2013). Altogether, hMSCs manufactured from microcarrier culture systems still meet the release criteria defined by ISCT, although the cells exhibit differences in surface markers, CFU-F, and lineage-specific gene expression.

### Therapeutic Potency: Differentiation Potential, Migration Ability, and Secretory Function

To date, more than 1000 completed or ongoing hMSC-based clinical trials have been reported (Tsuchiya et al., 2019), including bone and cartilage regeneration, graft-vs.-host disease, kidney injury, liver disease, myocardial infarction, and type I and II diabetes (Chen et al., 2013; Simaria et al., 2014). These clinical applications of hMSCs are attributed to unique stem cell properties: renewability of regeneration, capacity for multi-lineage differentiation, migration ability to inflammatory tissue, and secretion of anti-inflammatory and pro-angiogenic trophic factors (Aggarwal and Pittenger, 2005; Prockop et al., 2010; Wang et al., 2012; Bianco et al., 2013; Stroncek et al., 2014). However, most therapeutic characterizations are based on hMSCs expanded from planar culture system. Thus, it is necessary to characterize hMSCs manufactured from microcarrier culture, particularly in therapeutic efficacy which is generally indicated by differentiation potential, migratory ability, and secretory function.

#### Differentiation Potential

It is well-acknowledged that hMSCs are able to differentiate into osteoblasts, adipocytes, and chondrocytes *in vitro* using inducing factors in the medium (Pittenger et al., 1999). The tri-lineage differentiation capability is included in the release criteria of hMSCs by ISCT (Dominici et al., 2006). Beyond biochemical signaling, physical cues including gravity, adhesion geometry, surface elasticity, adhesion force, and fluid shear stress all contribute to lineage commitment and differentiation (Zayzafoon et al., 2004; Engler et al., 2006; Kilian et al., 2010; Yourek et al., 2010; Mathieu and Loboa, 2012). For bone and cartilage regeneration, high yield of differentiated cells from hMSCs within a short time frame is preferred. Many groups have reported that microcarrier culture improves hMSC osteogenic and chondrogenic differentiation potential *in vitro* or *in vivo* (Tseng et al., 2012; Goh et al., 2013; Shekaran et al., 2015, 2016; Lin et al., 2016; Gupta et al., 2018, 2019). For example, microcarrier-expanded hMSCs were reported to have considerably increased pellet size and DNA content, as well as higher production of glycosaminoglycan and collagen II per pellet, after 28-days chondrogenic differentiation, compared to those from planar culture (Lin et al., 2016). The microcarrier-expanded hMSCs were also found to have increased osteogenic gene expression, alkaline phosphatase activity, calcium deposition, and collagen I secretion compared to the planar control (Tseng et al., 2012). The enhancement of osteogenesis may arise from increased cytoskeletal tension and actomyosin contraction of hMSCs on microcarriers, which can be inhibited by latrunculin B and blebbistatin (Tseng et al., 2012). By contrast, lower gene expression of adipocyte markers, such as PPARγ2 was observed in microcarrier culture, which demonstrates that microcarrier culture may down-regulate adipogenic differentiation potential (Tseng et al., 2012). Indeed, dynamic microcarrier culture system could alter hMSC commitment and differentiation compared to planar culture.

#### Migration Ability

hMSCs demonstrate homing and migration ability to the injured or disordered tissues *in vivo* after administration. Thus, migratory capacity is an important indicator for hMSC-based therapies. While not many studies examined hMSC migratory ability after microcarrier expansion, some evidence of migratory ability changes can be found. It has been reported that microcarrier-expanded hMSCs have smaller size, and display higher CXCR4 expression in comparison with planer culture (Sun et al., 2010; Levato et al., 2015), suggesting that microcarrier-culture possibly enhanced hMSCs' migration ability. Further *in vitro* and *in vivo* examinations should be performed to elucidate this possibility.

#### Secretory Function

hMSCs also play an important role in secreting growth factors, chemokines, and cytokines to maintain the physiological environment and attenuate immunogenicity in their original niche (Aggarwal and Pittenger, 2005; Ren et al., 2008; Liang et al., 2014). It has been reported that microcarrier-expanded fetal hMSCs secreted higher levels of IL-6 (an immunomodulatory cytokine), IL-8 (a pro-angiogenic chemokine), and CXCL5 (a chemokine) than planar culture-expanded cells (Shekaran et al., 2015). Also, changes in secretome of hMSCs cultured on microcarriers facilitated neuroregulatory function and differentiation of neural progenitor cells *in vitro* and *in vivo* as compared to those from planar culture (Teixeira et al., 2016). Specific proteins involved in the central nervous system, such as cystatin C, glia-derived nexin, galectin-1, and pigment epithelium-derived factor, were upregulated in microcarrier systems as well. Similarly, after tumor necrosis factor-α and interferon γ stimulation, microcarrier-expanded umbilical cord-derived hMSCs exhibited higher secretion of VEGF, interleukin 1 receptor antagonist (IL-1ra), and stromal cell-derived factor 1-alpha (SDF-1α) compared with flask-expanded hMSCs. Likewise, amniotic membrane-derived hMSCs cultured in microcarrier bioreactors secreted more VEGF, basic fibroblast growth factor, macrophage colony-stimulating factor (M-CSF), nerve growth factor, monocyte chemotactic protein-1 (MCP-1), and hepatocyte growth factor than flask-cultured hMSCs (Hupfeld et al., 2014). Upregulated secretion of brain-derived neurotrophic factor and insulin-like growth factor 1 was also reported in microcarrier systems (Teixeira et al., 2016). Furthermore, functional studies with immune cells under inflammation indicated that MSCs cultured on microcarrier exhibited improved IDO activity and thus maintained their immunomodulatory potentials characterized by inhibition of T cell proliferation (Lawson et al., 2017; Das et al., 2019). Taken together, microcarrier culture likely promotes hMSC's therapeutic potency in immunomodulation and angiogenesis by altering their secretome profiles.

## The Microenvironment Change in Microcarrier-Based Bioreactors

The alteration of hMSC properties is most likely associated with the discernible differences in culture microenvironment between planar and microcarrier culture (Sart et al., 2013; Ma et al., 2016). This part is to elucidate the possible relationship between hMSC properties and bioprocessing parameters/microenvironment based on two factors: the adhesion surface and the hydrodynamics.

### Adhesion Surface: Discontinuous Surface, Convex Curvature, and Microcarrier Rigidity

The primary adhesion surface change is the substrate topography which means cells colonize on individual disconnected microcarriers with a convex curvature. Other properties of adhesion substrate including rigidity, roughness, porousness, coating, charge, hydrophilicity, and wettability may also influence cellular microenvironment.

#### Discontinuous Surface

Upon inoculation, the microenvironment in microcarrier culture immediately changes compared to planar culture. Microcarriers provide abundant accessible surface per unit volume while lack of a bridge between individual microcarriers impedes cell migration from bead to bead. In planar culture, however, migration can compensate non-homogeneous cell distribution at a certain level. Migration on microcarriers is completely different as cells can only migrate within the single bead under dynamic environment. Thus, simply counting cell attachment efficiency is not informative without considering the percentage of microcarriers with attached cells. Generally, inoculation cell density is determined by the cell-to-bead ratio which needs to reach the threshold of critical cell number per microcarrier (Hu et al., 1985). In literature, a seeding density of 5 cells per microcarrier is commonly used for hMSCs (Rafiq et al., 2013a). Initial colonization of microcarriers by cells theoretically can be estimated by Poisson distribution (Frauenschuh et al., 2007), and the expected percentage of occupied microcarriers at a cell-to-bead ratio of 5 is 99.3%. Due to discontinuous surfaces, the homogeneous cell attachment on microcarriers and initial percentage of colonized microcarriers are critical for maintaining culture microenvironment and preventing uneven distribution of cells and mass transfer limitations.

Even if perfect homogeneous cell attachment on microcarriers can be achieved, the natural heterogeneity of hMSC populations still exists (Phinney, 2007). These heterogeneous characteristics including proliferation rate, metabolic activity, and contact limit would result in different confluence on each microcarrier at harvesting. Some cells on microcarriers may reach the stationary phase, while other cells are still in the lag phase or exponential phase. In addition, the heterogeneity of microcarriers also contributes to the heterogeneity of hMSCs. For example, SoloHill® plastic microcarriers formed by cross-linked polystyrene have the size range of 125–212 μm in diameter, and 1.7 times of difference in diameter would result in 2.9 times of difference in surface area. Also, therapeutic potency may vary for cells harvested at different confluency (Lam et al., 2019). Thus, the right timing for harvest should ensure that a majority of microcarriers are ready.

#### Convex Curvature

**Figure 3:**
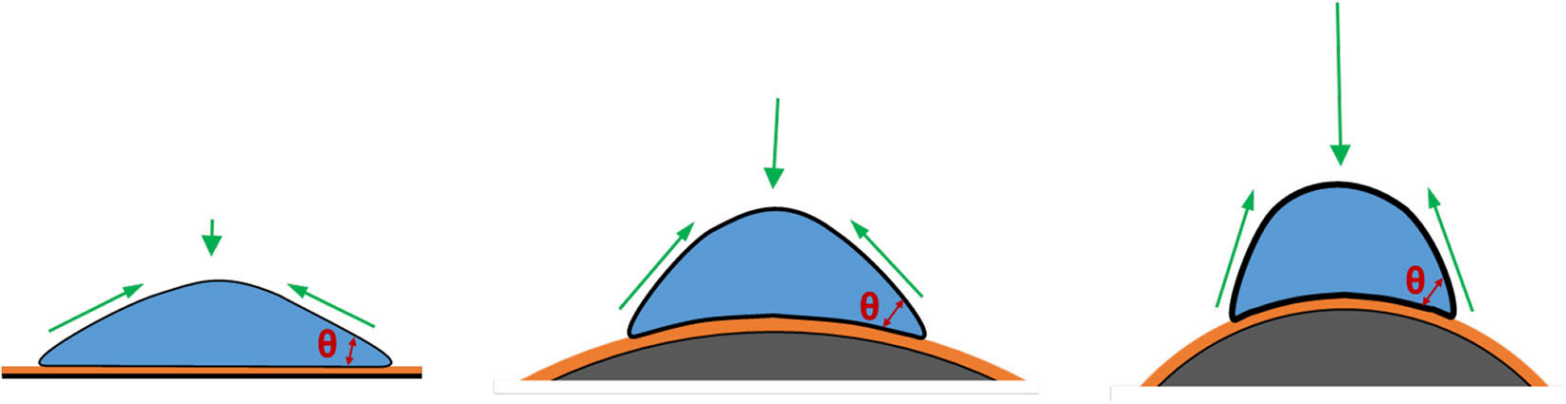
Effects of substrate curvature. Compared to cells cultured on the planar surface (Left), cells cultured on the convex surface (Middle and Right), like microcarriers, have a higher contact angle and thus under a higher mechanical force, against which cells develop more actin stress fibers, as a result of increased cytoskeletal tension. This trend shows more significant when the microcarriers are smaller.

Geometric cues can regulate cell fate and differentiation commitment in planar culture via the changes of mechanical microenvironment, leading to a reorganization of cytoskeletons and formation of myosin-generated contractility (Chen et al., 2003; Discher et al., 2005; Kilian et al., 2010). These geometric cues include the dimension, shape, and curvature of the culture surface. In microcarrier culture, the accessible surface area provided by a single microcarrier with a diameter of 100–300 μm is in the range of 0.03–0.28 mm^2^, which is much larger than the area for a spreading single cell (Figure 3). Therefore, the curvature would be the dominant factor in topographic cues. The curvature of a surface is defined as the reciprocal of the radius of the sphere fitting to the camber (Xu et al., 2011; Ueki and Kidoaki, 2015). The curvature of a microcarrier is ranged from 1/50 to 1/150 μm^−1^, and the level of the curvature is inverse to the microcarrier size.

To investigate the role of curvature, both microcarriers and planar surfaces should be made of the same materials with the same biophysical characteristics, and with cells cultured under the same flow condition. Indeed, it is difficult to isolate the effect of curvature during microcarrier suspension culture. Researchers have tried to use microprinting techniques to generate different geometric cues to address the influence of dimension and geometric shape on cytoskeleton distribution and mechanical contractility (Théry et al., 2006; James et al., 2008; Kilian et al., 2010; Théry, 2010), though it is still not the optimized model to mimic the underlying curvature on microcarriers. Recently, Werner et al. (2017) cultured hMSCs on the stereolithographic convex spherical structures with the curvature of 1/125, 1/175, 1/250, and 1/375 μm^−1^, which have the similar topography to semi-spheres, partially mimicking the curvature on microcarriers. The results reveal that cells on convex spherical structures have higher F-actin intensity and higher osteocalcin levels compared to cells on flat surfaces after 10-days expansion or osteogenic induction, and the enhancement becomes more significant when decreasing the convex diameters (Werner et al., 2017). The enhanced mechanical stress may be attributed to the push-force by the perinuclear actin cap to deform the nucleus (Werner et al., 2017). Based on the tensegrity theory, a numerical model was used by Vassaux to investigate the influence of biophysical environment on adherent cells on concave hemispheres (Ingber, 2003a). The results indicate that cells can experience higher mechanical microenvironment and become stiffer if the underlying substrate becomes more convex (Vassaux and Milan, 2017). Thus, higher underlying curvature from the smaller microcarrier size may contribute to higher mechanical stress and improved osteogenic differentiation. Another study was performed by culturing hMSCs on 4, 3, 2, 1.1 mm-, 900, 750, and 500 μm-diameter glass balls, which were half-embedded in polyacrylamide gels. hMSCs exhibited the increased gene expression of adipogenesis when cultured on the beads with the size in diameter equal or below 1.1 mm (Lee and Yang, 2017).

To measure cell contractility, cells were seeded on a microprinted surface covered by crowded micropillars. Through the direction and the level of localized deformation of the micropillar top, the contractile force can be calculated (Du Roure et al., 2005; Ghassemi et al., 2012; Trichet et al., 2012). To further understand the effect of the biophysical microenvironment in microcarrier culture, similar techniques should be developed and applied to the microcarrier-like curvature.

#### Microcarrier Rigidity

The biophysical cues, such as substrate elasticity, have been reported to influence hMSC lineage specification (Discher et al., 2005; Engler et al., 2006). hMSCs can be induced into osteogenic differentiation on a surface with the elasticity of 25–40 kPa, but into myogenic differentiation on a softer surface (8–17 kPa) and neurogenic differentiation on the softest surface (0.1–1 kPa). Consequent studies demonstrated that cells sense the stiffness of ECM via heterodimeric integrin receptors of α and β subunits through focal adhesions with involved proteins, including talin, paxillin, and vinculin (Liu et al., 2000). Followed by actin polymerization and elongation, the stress fibers are formed, which are induced by the RhoA-Rho-associated coiled-coil containing protein kinase (ROCK)- myosin light chain phosphatase (MLCP) signaling pathway responsible for cell skeletal tension along the edges (Galbraith et al., 2007). The integrin-mediated mechanotransducers, e.g., ROCK, activated by RhoA activity and influenced by cell adhesion and actin-myosin tension regulate hMSC lineage commitment through the mitogen activated protein kinase (MAPK)/the extracellular-signal-regulated kinase (ERK) and Yes-associated protein/transcriptional coactivator with PDZ-binding motif (TAZ) signaling pathways (McBeath et al., 2004; Bhadriraju et al., 2007; Dupont et al., 2011; Shih et al., 2011; Kim et al., 2014). The surface stiffness with the capacity of regulating hMSC differentiation is identified in planar culture, but whether the surface stiffness still has the same effects in microcarrier system, and whether it is possible to use the particular microcarrier stiffness to produce a specific lineage of hMSCs have not been well-investigated.

To date, the characteristics of microcarriers provided by the vendors usually only contain the information of size, composition, density, porosity, surface area, coating, and surface charge, but not the elasticity, rigidity, or stiffness. Most microcarriers are composed of a mixture of peptides or polymers, and the elasticity of these materials differs with the combination and concentration of molecules (i.e., degree of crosslinking) and the average length of polymers (i.e., degree of polymerization). For example, Lück et al. (2016) proposed a fabrication method of synthetic hydrogel microcarriers by telechelic poly(2-oxazoline) crosslinkers and methacrylate monomers, and the microcarrier rigidity can be regulated from 2 to 20 kPa in Young's modulus. Microcarrier rigidity can be measured by the high-resolution elasticity microscope (Cohn et al., 2000) with colloidal probe spectroscopy, an atomic force microscopy modified by gluing a micron-sized spherical force sensor to the end of cantilever (Kappl and Butt, 2002; Butt et al., 2005; Lück et al., 2016). To date, the influence of microcarrier rigidity on hMSC properties has not been well-investigated, possibly due to the confounded curvature effect on mechanical stress from the substrate. Hence, cells on microcarriers may not be as sensitive to the stiffness as they are on the flat surface.

### Hydrodynamics: Shear Stress and Collision and Aggregation

The flow in microcarrier culture provides dynamic environment capable of resuspending microcarriers for homogeneous mixing and induces non-homogeneous shear stress and microcarrier-microcarrier collisions, all of which have critical impacts on the extracellular microenvironment of hMSCs.

#### Shear Stress

In microcarrier culture, fluid flow plays a critical role in maintaining microcarriers in suspension and induces shear stress in culture microenvironment. Flush-mounted film probes (Dantec Dynamics, Germany) can be used to measure flow-induced shear stress, but have to be stuck on the wall (Kalmbach et al., 2011). Particle image velocimetry is able to generate comprehensive profiles of fluid shear stress and velocity in the spinner flasks (Ismadi et al., 2014). Computational fluid dynamics also offers an alternative approach to predict fluid shear stress (Jossen et al., 2016; Tsai et al., 2017). From these results, the maximum shear stress is 0.07–0.1 Pa at 25 rpm and 0.22–0.31 Pa at 60 rpm. Although only a small population of cells are exposed to the localized high shear stress and the volume-weighted mean of shear stress is very low (e.g., 0.0032 Pa at 25 rpm and 0.0067 Pa at 60 rpm), accumulated influence induced by shear stress is still significant on cell growth (Yuan et al., 2014; Jossen et al., 2016). Accordingly, alternative bioreactor designs have been developed to provide a low-shear stress environment, such as WAVE Bioreactor™ (GE Healthcare), BIOSTAT® CultiBag RM bioreactor (Sartorius), and Vertical-Wheel™ bioreactor (PBS Biotech) (Timmins et al., 2012; Sousa et al., 2015; Jossen et al., 2016; da Silva et al., 2019).

When flow-induced physical force directly acts on the cells, the cells may respond in several possible ways. For example, Ingber proposed a theory of tensegrity that a cell is structured by a hierarchical framework of cytoskeletons (Ingber, 2003a,b). Thus, the external forces acting at any point of the cell would be translated into the whole internal cell structure to revoke the force effect by cytoskeletal reorganization. Another possibility is that shear stress mechanically influences the integrins that connect ECM and cytoskeletons through the focal adhesion and the anchorage proteins to mechanotransduce the biomechanical stress into biochemical signals (Ingber, 2003b). Moreover, the cell membrane become more fluidized to change membrane composition and ion channels for the mechanotransduction pathways under elevated fluid shear stress (Barakat et al., 2006). These responses expedite hMSCs' commitment to osteogenic lineage by regulating opening and closing of membrane ion channels to increase intracellular Ca^2+^ concentration, and reorganizing cytoskeleton which activates focal adhesion kinase (FAK)/ERK1/2 pathways to trigger Runx2 and AP-1 and initiate the transcripted osteogenic differentiation (Liu et al., 2010). In addition to osteogenic differentiation, microcarrier culture has also been reported to improve chondrogenic differentiation (Lin et al., 2016). hMSCs undergo chondrogenic differentiation normally requiring cells clustering into spheroids that provide the unique mechanical microenvironment (Sart et al., 2014b; Tsai et al., 2015). Thus, shear stress in microcarrier culture reshapes the microenvironment for hMSCs to exhibit lineage specificity.

#### Collision and Aggregation

In bioreactors, microcarrier-impeller and microcarrier-microcarrier collisions occur frequently when mixing microcarriers in suspension. Microcarrier concentration and agitation speed determine the collision frequency and microcarrier kinetic energy, respectively.

Increased microcarrier concentration can increase the volumetric production at a lower cost, and higher cell culture density can induce more concentrated autocrine and paracrine factors which accelerate cell growth and facilitate cell function. However, high microcarrier concentration may promote collision frequency and damage cell growth. For example, addition of Sephadex G-50 beads (Pharmacia) was used to change the collision frequency. Severely decreased cell growth was observed at high collision frequency, which may detach cells from microcarriers (Croughan et al., 1988). Furthermore, increased microcarrier concentration normally requires frequent medium change and results in nutrient limitations or toxic by-product accumulation due to high cell density (Chen et al., 2015), which can be overcome by a perfusion culture system.

Agitation speed associated with microcarrier kinetic energy has been reported to influence hMSC expansion, and the optimal cell growth was observed at 49 rpm in a 100 mL spinner flask (Yuan et al., 2014; Jossen et al., 2016). Jossen et al. demonstrated that hMSC expansion significantly decreased when the agitation speed reached above 90 rpm (Jossen et al., 2016). Besides the shear stress effect, another possible explanation is the increased physical collisions due to the elevated kinetic energy and collision frequency (Yuan et al., 2014). The agitation speed of above 90 rpm may reach the threshold of microcarrier kinetic energy for initiating cell death (Jossen et al., 2016). The highest velocity at the tips of impeller can be calculated by agitation speed and impeller diameter (Ismadi et al., 2014; Odeleye et al., 2014). For example, in a 100 mL spinner flask with a standard impeller of 4 cm in diameter, the tip speed is 0.19 m/s at 90 rpm.

The accumulated collision impact likely leads to cell death and phenotypic alteration. Although the damage from collisions depends on the frequency and the intensity, it is difficult to isolate the shear stress effect. Moreover, cell concentration or cell coverage on microcarriers can also impact collision damage at the late stage of cultivation. Clusters bridged by cells may form due to the microcarrier-microcarrier collisions and non-proper mixing, especially when cells are confluent (Cherry and Papoutsakis, 1988; Caruso et al., 2014). Once multiple-microcarrier clusters are formed, cells tend to migrate to interstitial space between microcarriers, and multi-cellular aggregation has been well-known to promote cell differentiation, migration, and secretion (Sart et al., 2014b; Cesarz and Tamama, 2016; Zhang et al., 2016). Nevertheless, cells in the clusters may endure the limitation of mass transfer since diffusion is dominant over convection. Besides, shear stress randomly tears apart bridged microcarriers and may further provoke cell death (Cherry and Papoutsakis, 1988; Takahashi et al., 2017). Therefore, the microcarrier concentration and the agitation speed along with culture time need to be optimized to avoid severe collision damage and oversized clusters (Chen et al., 2015).

## Conclusion

To expand hMSCs under cGMP compliant regulations, advanced biotechnologies have been developed, such as multi-layer vessels, fixed/packed bed bioreactors, hollow fiber bioreactors, and microcarrier suspension bioreactors. However, there is no guarantee that hMSC therapeutic potency is well-preserved, owing to the fundamental change in culture microenvironment. As a result, standardized characterization of manufactured hMSCs from different bioprocesses is necessary to certify their therapeutic potency for specific disease models. This article focuses on the microcarrier bioreactor systems and discusses the influences of bioprocessing parameters (e.g., agitation speed, heterogeneous shear stress exposure, microcarrier size, rigidity, adhesive force, coating charge, and cell-cell collision and aggregation) on the differentiation, migratory ability, and secretory function of manufactured hMSCs. Based on literature, microcarrier culture has been reported to enhance hMSC osteogenic and chondrogenic differentiation but impair their adipogenic differentiation compared to planar culture. Moreover, the improved migration and secretion abilities suggest microcarrier culture may augment hMSC therapeutic potency in immunomodulation, angiogenesis, and neural differentiation. Overall, microcarrier culture in bioreactors provides the possibility to scale up hMSC production and regulates hMSC therapeutic properties for clinical applications. Future studies should focus on improving the robustness of hMSC biomanufacturing system as well as engineering hMSCs with desired stem cell properties.

## Author Contributions

A-CT wrote the majority of the manuscript. RJ and XC revised the manuscript. XY and YL conceived the whole study, revised, and finalized the manuscript.

## Conflict of Interest

The authors declare that the research was conducted in the absence of any commercial or financial relationships that could be construed as a potential conflict of interest.
